# Dynamics of PCV2 and PCV3 in the Serum and Oral Fluids of Pigs After PCV2 Vaccination in a Commercial Farm

**DOI:** 10.3390/vaccines12121318

**Published:** 2024-11-26

**Authors:** Jesús Hernández, Alexanda Henao-Díaz, Mónica Reséndiz-Sandoval, Angel Cota-Valdez, Verónica Mata-Haro, Luis G. Gimenez-Lirola

**Affiliations:** 1 Laboratorio de Inmunología, Centro de Investigación en Alimentación y Desarrollo, A.C., Hermosillo 83304, SON, Mexico; mresendiz@ciad.mx; 2 Grupo Bachoco, Unidad de Negocios Cerdo, Celaya 38000, GTO, Mexico; yulyalex@gmail.com (A.H.-D.); angedlcota@hotmail.com (A.C.-V.); 3 Laboratorio de Microbiología e Inmunología, Centro de Investigación en Alimentación y Desarrollo, A.C., Hermosillo 83304, SON, Mexico; vmata@ciad.mx; 4 Department of Veterinary Diagnosis and Production Animal Medicine, College of Veterinary Medicine, Iowa State University, Ames, IA 50011, USA

**Keywords:** PCV2, PCV3, co-infection, vaccine, antibodies, oral fluids

## Abstract

Objectives: This study investigated the dynamics of porcine circovirus type 2 (PCV2) and PCV3 on a commercial farm following PCV2 vaccination. Methods: Serum samples from 35 pigs, starting at 3 weeks of age, were collected weekly until 21 weeks of age. Oral fluids from six pens of pigs of the same age were also analyzed. Viral DNA was assessed in pooled sera and individual oral fluid samples, while antibodies (IgG and IgA) were measured in the serum and oral fluids. Productive parameters, including weekly mortality and cumulative mortality, were evaluated. Results: The results revealed that PCV2 and PCV3 co-infection was detected in pigs at 8 weeks of age, with PCV3 being detected in oral fluids two weeks earlier. PCV3 DNA was detected in oral fluids at 4 weeks of age. PCV2 IgG antibodies in the serum increased gradually after vaccination, peaking at 7 weeks of age, then declined and stabilized until 21 weeks of age. PCV3 IgG antibodies fluctuated early but were uniformly positive after 13 weeks of age. In oral fluids, PCV2 IgG and IgA antibodies showed a strong response only at 3 and 23 weeks of age. In contrast, a strong and consistent IgG response was observed in oral fluids in the absence of PCV2 and PCV3 co-infection of pigs at 3 to 11 weeks of age. The farm’s productive parameters remained stable throughout the study. Conclusions: These findings suggest that PCV2 and PCV3 co-infection, along with high PCV3 detection levels in serum and oral fluids, may have an impact on the efficacy of PCV2 vaccination.

## 1. Introduction

The *Circoviridae* family affects several species, including pigs, with porcine circoviruses (PCVs) being of significant concern to the swine industry [1]. These viruses possess a small, circular virion and a genome composed of single-stranded DNA of approximately 2 kb. In pigs, four distinct species of PCV have been identified: PCV1, PCV2, PCV3, and PCV4 [2]. Among these, PCV1 and PCV4 are considered nonpathogenic, although further research is needed to fully understand the potential impact of PCV4 on swine health and production [2].

PCV2, in contrast, is a major pathogen with a global distribution recognized for causing PCV2-associated diseases (PCVADs). PCVAD encompasses a range of clinical presentations, including systemic disease, pneumonia, reproductive disorders, respiratory problems, and subclinical infections, all of which have substantial economic consequences for the swine industry. Although effective vaccines can mitigate clinical signs and reduce viremia, they do not prevent infection. Nevertheless, vaccination remains an essential tool, particularly in reducing reproductive losses and minimizing the impact of PCVAD on growth and respiratory function in pigs [3].

Under experimental conditions, PCV2 DNA can be detected in oral fluids as early as 2 days post-infection, with detection persisting for up to 98 days post-infection [4]. Similarly, the detection of PCV2 DNA in serum follows a similar pattern [5]. Immunoglobulin analysis of IgG, IgA, and IgM in oral fluids revealed a gradual increase in IgG, IgA, and IgM antibodies, reaching peak levels between 65 and 75 days post-infection, followed by a gradual decline. Despite this decline, antibody levels remained detectable until day 98 post-infection, as observed under the experimental conditions reported by Prickett et al. (2011). Notably, a similar profile was observed for serum IgG [4]. 

Most PCV2 vaccines elicit a robust and long-lasting antibody response that is detectable in serum and oral fluids. This vaccine-induced response mirrors that observed after experimental infection, although it can be more pronounced following a booster dose. However, as stated previously, while this antibody response reduces viremia, it does not prevent infection [6].

Like PCV2, PCV3 appears to be a ubiquitous virus that has been reported in many countries and detected in symptomatic and asymptomatic pigs [7,8,9,10,11,12,13,14,15]. The primary clinical manifestations of PCV3 include respiratory disease in piglets and growing pigs, as well as reproductive failure in sows. Additionally, PCV3 has been linked to neurological or digestive disorders. In experimental infection studies involving cesarean-derived colostrum-deprived pigs, PCV3 DNA was detected as early as 3 days post-infection, with viremia persisting until the end of the study on day 35 [16]. Other studies have reported that PCV3 DNA persists for up to 5 months in naturally infected wild boars [17].

In experimentally infected pregnant gilts, viremia became evident two weeks post-infection and persisted through the end of the study (day 91). Most piglets born to these gilts are viremic at birth and remain positive for PCV3 DNA until weaning [18]. While several studies have examined the prevalence of PCV3 DNA in serum, tissues, and oral fluids under field conditions [19], few have focused on seroprevalence [15,20,21,22]. Experimentally, PCV3 IgG is detectable as early as 7 days post-infection, with a slow increase followed by a rapid decline [16]. To date, there are no studies that have evaluated antibody dynamics following natural or experimental PCV3 infection, although some research has identified IgG antibodies in both symptomatic and asymptomatic pigs [15].

Several authors have documented co-infection of PCV2 and PCV3, with both viruses being detected in serum, tissues, and oral fluids [19,23]. A recent study evaluated the presence of PCV2 and PCV3 in pooled tissue samples from piglets and oral fluids from grow-finish pigs across 16 farms in Germany and Austria [24]. In piglets, PCV3 DNA was more prevalent than PCV2 DNA was, whereas in the grow-finish stage in pigs, the prevalence of both viruses was similar (mean values for PCV2: 31.1, 29.0, 28.6, and 29.2; and for PCV3: 30.1, 30.1, 30.3, and 31.9). The authors attributed the lower prevalence of PCV2 DNA in piglets to PCV2 vaccination, which is known to reduce viremia [24]. In the grow-finish pigs, the Ct values for PCV2 and PCV3 at 6, 12, 16, and 20 weeks of age were comparable.

Despite numerous studies reporting co-infection, there is currently no evidence to suggest that PCV3 interferes with PCV2 vaccination, enhances PCV2 pathogenicity, or increases susceptibility to other pathogens. Woznail et al. (2019) investigated the effect of PCV3 infection on PCV2 vaccination and reported no adverse interactions [25]. However, this study revealed a low co-infection rate between PCV2 and PCV3. Further research with higher rates of co-infection is needed to confirm these findings.

The dynamics of anti-PCV3 IgG antibodies under field conditions remain poorly understood, and it is unclear whether PCV2 and PCV3 co-infection affects antibody responses or viremia. While anti-PCV2 antibody analysis under field conditions provides insight into the pigs’ response to vaccination, it does not indicate protection. Nonetheless, antibody analysis in serum and oral fluids provides valuable information about the overall immune response. In the case of PCV3, this approach can provide information on antibody persistence and viral circulation. Examining the dynamics of both anti-PCV2 and anti-PCV3 antibodies, alongside viral leads, across various locations could yield important data on the effects of co-infection within farm environments. This study aimed to evaluate IgG antibody levels in serum and IgG and IgA antibody levels in oral fluids against both PCV2 and PCV3.

## 2. Materials and Methods

### 2.1. Farms

A follow-up study was conducted on a farm located in northwestern Mexico free of porcine reproductive and respiratory syndrome virus (PRRSV) and *Mycoplasma hyopneumoniae* and had a history of PCV3 detection and an established PCV2 vaccination program using a commercial subunit vaccine based on ORF2. The conventional pigs were located at Site 1, where 650 sows were housed. They were initiated into a PCV2 vaccination protocol in which piglets received a single dose at 21 days of age; the gilts received two doses before insemination, and sows received one dose at 14 days post-farrow. Additionally, mass vaccination was performed at Site 1 6 weeks after the follow-up study was initiated.

**Figure 1:**
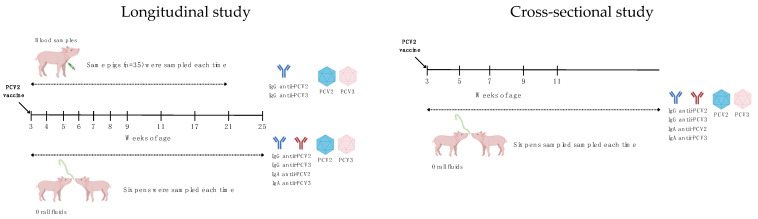
Experimental design. This study comprised two separate investigations: a longitudinal study and a cross-sectional study. The longitudinal study evaluated PCV2 and PCV3 viremia, as well as the presence of IgG antibodies against PCV2 and PCV3 in the serum. Additionally, viral loads and IgG and IgA antibodies against PCV2 and PCV3 in oral fluids were quantified in pens of pigs aged 3 to 25 weeks. The cross-sectional study measured viral loads and IgG and IgA antibodies against PCV2 and PCV3 in oral fluids from pens of pigs aged 3 to 11 weeks.

Piglets were weaned at 23 days and subsequently moved to Site 2 and then to Site 3 after 7 weeks. A cohort of 35 pigs were selected for weekly blood sampling from 3–21 weeks of age, with pen-based oral fluids collected weekly. After 16 weeks, cross-sectional oral fluid sampling was conducted, and samples were collected from pigs aged 3, 5, 7, 9, and 11 weeks (Figure 1).

### 2.2. Samples

Blood samples were collected from 35 three-week-old pigs before weaning and PCV2 vaccination. Each pig was individually ear-tagged and sampled weekly until nine weeks of age. Blood samples were subsequently collected thrice at 4-week intervals until 21 weeks of age. Oral fluids (*n* = 6) were collected from three pens, with two samples per pen, starting when the pigs were three weeks old and continuing until 21 weeks of age. Blood and oral fluids were collected simultaneously and stored at −80 °C until processing. During the second sampling, oral fluids (*n* = 6) were collected from the three pig pens at 3, 5, 7, 9, and 11 weeks of age and stored at −80 °C until processing.

### 2.3. ELISA

ELISAs were conducted as previously described, with modifications [15]. PCV3 and PCV2 capsid proteins (CAP), both at a concentration of 2 µg/mL, were diluted in coating buffer (ImmunoChemistry Technologies, Davis, CA, USA). A volume of 100 µL was added to Maxisorp ELISA microwell plates (Nunc, Thermo Fisher Scientific, Waltham, MA, USA) and incubated overnight (14–16 h) at room temperature (25 °C). Following the blocking step (General Block Buffer, ImmunoChemistry Technologies, Davis, CA, USA), the serum samples were diluted 1:100 with General Sample Diluent (ImmunoChemistry Technologies) and incubated for 30 min with slow agitation. The oral fluids were diluted 1:1 with phosphate-buffered saline (PBS) pH 7.4 and incubated overnight at 4 °C. The plates were subsequently washed with PBS containing 0.1% Tween 20 (Cat. No. 1610781; Bio-Rad, Hercules, CA, USA) and 50 µL of goat anti-porcine IgG-HRP (Polyclonal; Cat. No. 6050-05; SouthernBiotech, Birmingham, USA) was added and incubated for 30 min. The reaction was developed by adding 50 µL of 3,3′,5,5′-tetramethylbenzidine (TMB; Immunochemistry, Davis, CA, USA) and stopped with 50 µL of 1 M H_2_SO_4_. The plates were then read via an automated spectrophotometer (Thermo Scientific Multiskan FC Microplate Photometer, Temecula, CA, USA) at an optical density (O.D.) of 450 nm. Each plate included a positive control (a pool of sera from PCV3- or PCV2-infected swine), a negative control (a pool of sera from colostrum-deprived pigs) and blanks, all in duplicate. The results are presented as the O.D. values after the absorbance of the blanks were subtracted.

### 2.4. Real-Time PCR

DNA extraction from serum and oral fluid samples was conducted using the QIAamp DNA Mini Kit (Qiagen, Hilden, Germany) following the manufacturer’s instructions. The serum samples were pooled into groups of five, and the oral fluids were analyzed individually. Quantitative real-time PCR (qPCR) was used to detect PCV3 or PCV2 ORF2, as previously described [15]. The qPCRs were set up in a 25 μL volume comprising 150 nM of each primer, 10 μL of Brilliant III Ultra-Fast SYBR Green qPCR Master Mix (Agilent Technologies, Cedar Creek, TX, USA), 4 μL of DNA, and 10.25 μL of nuclease-free water. Amplification was performed using the StepOne Real-Time PCR system (Applied Biosystems, Austin, TX, USA) under the following cycling conditions: initial denaturation at 95 °C for 3 min, followed by 40 cycles of 94 °C for 5 s, 60 °C for 10 s, and 72 °C for 5 s. Samples with Ct values < 35 were considered positive.

### 2.5. Statistical Analysis

Data normality was assessed using the Shapiro–Wilk and Kolmogorov–Smirnov tests. While some groups showed a normal distribution, others did not. Consequently, a non-parametric approach was applied for further analysis. Differences in PCV2 and PCV3 IgG antibody levels were evaluated using the Kruskal–Wallis test, followed by Dunn’s test for multiple comparisons. All analyses were conducted with a significance level of 0.05 using GraphPad PRISM software (version 10.4.0).

## 3. Results

### 3.1. Productive Performance

**Figure 2:**
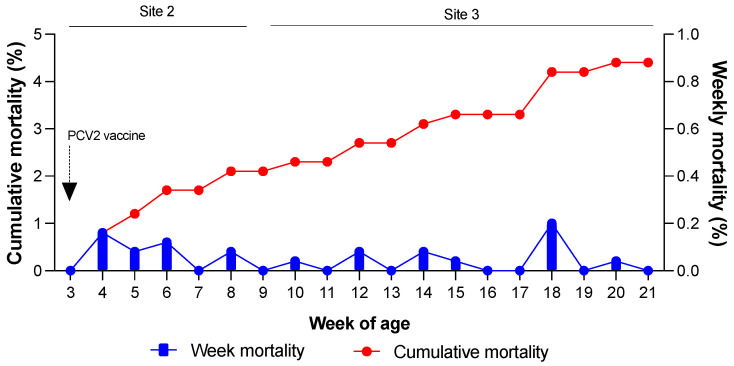
Cumulative mortality rates at Sites 2 and 3. Pigs were vaccinated against PCV2 at 3 weeks of age.

Weekly and cumulative mortality rates were evaluated to determine the production parameters of the farms (Figure 2). On Farm 1, Site 2 recorded a cumulative mortality of 2.1%, whereas Site 3 had a cumulative mortality of 2.6%. The maximum allowable mortality rates for Sites 2 and 3 were 2% and 4%, respectively. As shown in Figure 2, the observed mortality rates fell within the acceptable range for the farm. No signs of wasting, poor body condition, coat abnormalities, or respiratory distress were observed in the pigs. 

Week mortality was evaluated using the following formula:

$$Week\;mortality=(100)\times (\frac{No.\;of\;dead\;pigs\;in\;a\;week}{No.\;of\;live\;pigs\;in\;the\;previous\;week})$$

The cumulative mortality was evaluated using the following formula:

$$Cumulative\;mortality=(100)-(\;\frac{No.\;of\;live\;pigs\times 100}{No.\;total\;of\;pigs\;at\;week\;3})$$

### 3.2. Evaluation of PCV2 DNA and PCV3 DNA in Serum and Oral Fluids

Viral DNA was analyzed in serum and oral fluid samples collected from pigs aged 3 to 21 weeks. A total of 35 serum samples were pooled into groups of five and analyzed weekly for seven weeks, followed by monthly analysis for three consecutive months. Individual pigs were consistently identified, and the same animals were sampled throughout the study, as previously described. At 21 weeks of age, the pigs were relocated to a new site; however, samples were still collected from pigs of the same age cohort.

Table 1 and Table 2 present the analysis of PCV2 DNA in the serum and oral fluid samples, respectively. For the serum samples, two pools tested positive at 3 weeks of age. The samples were subsequently negative for four consecutive weeks before turning positive from 8 to 21 weeks of age, with the exception of some pools at 13 weeks of age, which were negative. The pools from 21-week-old pigs presented the lowest Ct values (GM 29.77; CI 28.43–31.16). In the case of oral fluids, samples collected between 3 and 5 weeks of age were negative. However, starting at 6 weeks of age, all the oral fluid samples were positive. Like the serum samples, the oral fluids collected at 21 weeks of age presented the lowest Ct values (GM 28.93; CI 27.48–30.47).

Table 3 and Table 4 present the analysis of PCV3 DNA in the serum and oral fluid samples, respectively. At least one positive pool was detected at each collection point. Only one pool tested positive for serum samples between 3 and 6 weeks of age. By 8 weeks of age, all pools were positive. Unlike those for PCV2, the lowest Ct values for PCV3 were observed at 8 weeks of age (GM 29.89; CI 25.62–28.22) and 21 weeks of age (GM 26.86; CI 26.22–27.52). With respect to oral fluids, only samples from 3-week-old pigs were negative. Low Ct values were obtained in pigs at 7, 8, and 9 weeks of age, with the lowest Ct occurring at 8 weeks of age (GM 26.28; CI 22.80–30.29).

### 3.3. Dynamics of the IgG Antibody Response Against PCV2 and PCV3 in Serum Samples

**Figure 3:**
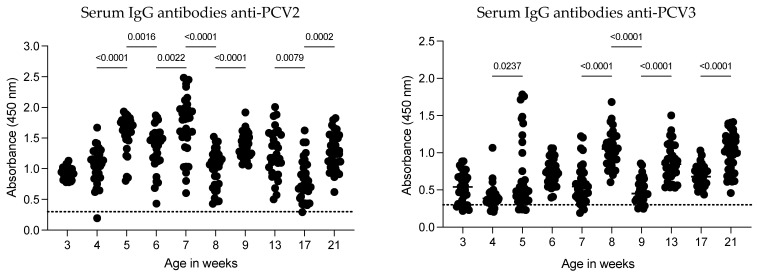
Serum IgG antibodies against PCV2 and PCV3. Serum samples were collected weekly from 35 pigs aged 3 to 21 weeks and tested for IgG antibodies against the CAP protein of PCV2 and PCV3. Each data point represents an individual pig, with the dotted line indicating the test cutoff. The results are expressed as the absorbance at 450 nm. Significant differences between weeks are shown above the data points.

The dynamics of IgG antibodies against PCV2 in serum were evaluated weekly in the same pigs from 3 to 21 weeks of age, with the last three evaluations conducted monthly (Figure 3). Except for two pigs (at weeks 4 and 17), all the animals showed IgG antibodies against the CAP protein of PCV2. The highest antibody levels were observed at 7 weeks of age, after which the antibody levels gradually increased. A significant decrease in anti-PCV2 IgG levels occurred at 8 weeks of age (GM 1.618; 1.437–1.823 to GM 0.9409; 0.8267–1.071; *p* < 0.0001). Following this decrease, antibody levels increased over the subsequent weeks until another reduction was observed at 17 weeks of age. However, the antibody levels did not return to the peak observed in pigs at 7 weeks of age. These findings suggest that anti-PCV2 IgG antibody dynamics can fluctuate as pigs age on commercial farms and that certain factors may contribute to reductions in anti-PCV2 antibody levels.

The dynamics of anti-PCV3 IgG antibodies were low at 3 and 4 weeks of age, with some pigs testing negative (5 out of 35 and 6 out of 35, respectively). A slight increase in antibody levels was observed at 5 weeks of age, with no significant changes at 6 and 7 weeks of age. However, maximal levels of anti-PCV3 IgG antibodies were detected at 8 weeks of age, followed by a decrease at 9 weeks of age. From 13 to 21 weeks of age, all pigs were positive for anti-PCV3 IgG antibodies. Overall, anti-PCV3 antibody levels were lower than anti-PCV2 antibody levels.

### 3.4. Dynamics of IgG and IgA Antibody Responses Against PCV2 and PCV3 in Oral Fluids

**Figure 4:**
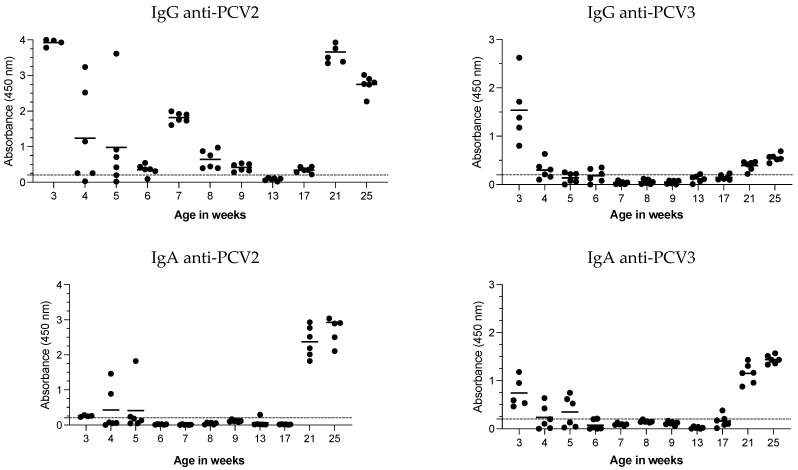
PCV2 and PCV3 IgG and IgA antibodies in oral fluids. Oral fluid samples were collected weekly from six pens of pigs aged 3 to 21 weeks and tested for IgG and IgA antibodies against the CAP protein of PCV2 and PCV3. Each data point represents an individual oral fluid sample, with the dotted line indicating the test cutoff. The results are expressed as the absorbance at 450 nm.

To better understand the humoral response to PCV2 and PCV3, both IgG and IgA antibodies against these viruses were evaluated in oral fluids, alongside serum samples, from 3 to 25 weeks of age (Figure 4).

For PCV2, pigs presented high levels of anti-PCV2 IgG in the oral fluids at 3 weeks of age. However, the IgG response varied from 4 to 5 weeks of age. At 6 weeks of age, the IgG levels were low but increased at 7 weeks of age. The response remained low or negative from 8 to 17 weeks of age and then increased at 21 weeks of age to levels similar to those observed at 3 weeks of age, with a slight decrease at 25 weeks of age. In contrast, the IgA levels were low or negative at 3 weeks of age, variable at 4 and 5 weeks of age, and remained negative from 6 to 17 weeks of age. Like those of IgG, anti-PCV2 IgA levels were high at 21 and 25 weeks of age.

For PCV3, the IgG response was high at 3 weeks of age but became variable (low/negative) from 5 to 6 weeks of age and was mostly negative from 7 to 17 weeks of age. Positive IgG responses were observed again at 21 and 25 weeks of age, although at low levels. The IgA response of IgA mirrored that of IgG: it was positive at 3 weeks of age, variable at 4 to 5 weeks of age, negative from 6 to 17 weeks of age, and positive again at 21 and 25 weeks of age.

### 3.5. Dynamics of IgG and IgA Antibody Responses Against PCV2 and PCV3 in Oral Fluids During the Cross-Sectional Analysis

**Figure 5:**
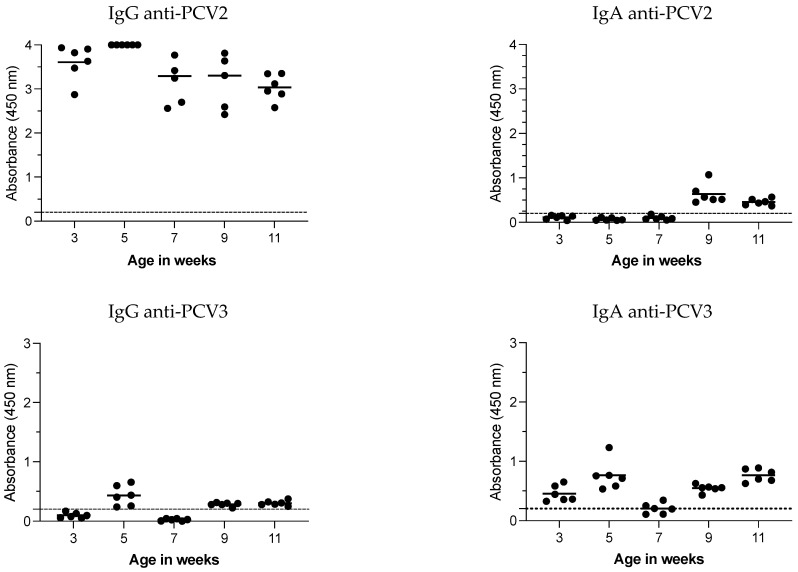
PCV2 and PCV3 IgG and IgA antibodies in oral fluids: cross-sectional study. Six oral fluid samples were collected weekly from pens of pigs aged 3 to 11 weeks and evaluated for the presence of IgG and IgA antibodies against the CAP protein of PCV2 or PCV3. Each data point represents an individual oral fluid sample, with the dotted line indicating the test cutoff. The results are expressed as the absorbance at 450 nm.

Sixteen weeks after mass vaccination (when the follow-up study was concluded), a cross-sectional analysis of IgG and IgA responses in oral fluids from pigs aged 3, 5, 7, 9, and 11 weeks (Figure 5) was performed. A high and consistent PCV2 IgG response was observed at all ages, whereas IgA was undetectable from 3 to 7 weeks but became positive at 9 and 11 weeks. For PCV3, IgG was detected at 5, 9, and 11 weeks but undetected at 3 and 7 weeks. In contrast, PCV3 IgA antibodies were consistently detected across all ages assessed.

### 3.6. Dynamics of PCV2 and PCV3 DNA in Oral Fluids During the Cross-Sectional Analysis

During the cross-sectional study, PCV2 and PCV3 DNA were undetectable in oral fluids from pigs aged 3 to 9 weeks. However, positive results for both viruses were observed at 11 weeks of age, although with Ct values > 32, indicating low viral loads (Table 5).

### 3.7. Monitoring of Piglets Born from a PCV3-Positive Sow

To evaluate the persistence of viral DNA under farm conditions, five piglets born from PCV3-positive and PCV2-negative sows were monitored weekly from 3 to 9 weeks of age and monthly thereafter until 17 weeks of age (Table 6 and Table 7). The results showed that all piglets tested negative for PCV2 from 3 to 7 weeks of age and positive from 8 to 9 weeks of age, with only one piglet remaining positive at 17 weeks of age. One piglet (pig 36) was consistently positive for PCV3 from 3 weeks of age until 17 weeks of age. Another piglet (Pig 38) was positive with low viremia from 5 to 17 weeks of age. The remaining pigs were positive from 7 weeks of age until the end of the experiment.

## 4. Discussion

This study evaluated the dynamics of PCV2 and PCV3 co-infection on a commercial farm, focusing on their impact on farm productivity and the antibody response to PCV2 vaccination. Our findings indicate that while co-infection with PCV2 and PCV3 may affect the presence of antibodies in oral fluids, it does not significantly alter productive parameters such as weekly and cumulative mortality under the studied conditions.

PCV3 DNA was detected earlier than PCV2 DNA in both serum and oral fluids. PCV3 DNA was detected in some serum pools of pigs as early as 3 weeks of age, persisting in all the serum pools until 21 weeks of age. Notably, pigs at 8 and 21 weeks of age presented Ct values of 25 to 27, which have been associated with disease caused by PCV3 [26]. In contrast, PCV3 DNA in oral fluids was first detected at 4 weeks of age, with pigs aged 7 to 9 weeks of age showing Ct values below 30. These findings suggest that PCV3 shedding may occur first in oral fluids before becoming detectable in serum, indicating that 7–9 weeks of age might be a critical period of peak viral excretion. These results emphasize the diagnostic importance of oral fluid sampling for the early detection of PCV3 [27].

Moreover, PCV3 can circulate intermittently in pigs, with viral shedding continuing at least until 21 weeks of age. Compared with PCV2 DNA detection, co-infection in serum started at 8 weeks of age, with the lowest Ct values (Ct of 28) occurring at 8 and 21 weeks of age. In oral fluids, PCV2 DNA was detected two weeks earlier than in serum, following a similar pattern to that of PCV3. These findings indicate that PCV2 and PCV3 perform differently in serum and oral fluids, with both viruses being detectable earlier in oral fluids (6 weeks of age) than in serum (8 weeks of age).

Five pigs born from a PCV3-positive sow were monitored from 3 to 17 weeks of age. One pig exhibited persistent viremia throughout the observational period, with Ct values < 30, except at 7 weeks of age. This finding is consistent with previous studies reporting prolonged viremia caused by PCV3 [17] and suggests that PCV3 infection may originate during maternity, spreading among pigs during weaning. This hypothesis could explain why most oral fluid samples were positive shortly after weaning and were all positive by 7 weeks of age.

No significant effects of PCV2 and PCV3 co-infection on farm productivity were observed, with weekly and cumulative mortality rates remaining within acceptable limits established by the producer. These results indicate that, under the specific conditions of this field study, co-infection did not increase mortality, even in pigs whose Ct values were less than 30 for PCV3 or PCV2. Although PCV3-related systemic disease has been described in pre- and post-weaning pigs [28], mortality in pigs has not been widely associated with PCV3, unlike sow mortality. PCV3 viremia in sows has been linked to reproductive failure, including abortion and perinatal mortality. Our findings are consistent with previous studies reporting that mortality in pigs is uncommon in cases of PCV3 [28,29].

Different reports have described the dynamics of PCV2 antibodies in experimental infections, vaccine-controlled experiments, and field vaccination experiments [4,5]. Some of these studies reported a robust serum IgG response to PCV2 following vaccination, along with detectable IgA antibodies in oral fluids. Our results partially support these findings. We observed an increase in serum IgG antibodies against PCV2 post-vaccination, with a peak at 7 weeks of age, but the response decreased in pigs aged 8 weeks and older. Similarly, PCV3 IgG antibodies showed a variable response, with some pigs testing negative at the earliest ages, whereas all pigs tested positive by 13 weeks of age. No previous studies have described the on-farm dynamics of PCV3 antibodies; however, our data suggest that this pattern might be common on farms lacking a PCV3 vaccination plan and with PCV3 viremia.

In oral fluids, a robust IgG response to PCV2 was observed in pigs at 3, 21, and 25 weeks of age, with a more erratic response occurring between 4 and 7 weeks of age. Pigs aged 9, 13, and 17 weeks tested negative or positive but close to the cutoff. In terms of IgA antibodies, a strong response against PCV2 was detected only in pigs aged 21 and 25 weeks. These findings differ from those of previous reports, which described strong IgG responses in oral fluids following PCV2 vaccination. A second analysis was performed to verify the accuracy of the immunoassay and further assess whether co-infection could interfere with the results. This subsequent investigation utilized a cross-sectional study design in which oral fluid samples from pigs aged 3, 5, 7, 9, and 11 weeks were evaluated. Interestingly, in this analysis, where pigs were negative for PCV2 and PCV3 (indicating no virus circulation), a robust PCV2 IgG response was observed in pigs aged 3, 5, 7, 9, and 11 weeks. These findings suggest that high PCV3 viremia may be related to the reduced IgG response in oral fluids at an early age.

The IgG and IgA responses against PCV3 in oral fluids followed a similar pattern, with detectable responses at 3 weeks of age and between 21 and 25 weeks of age. However, the absence of detectable IgG antibodies in pigs with viral excretion in oral fluids cannot be fully explained. The IgG antibodies detected at 3 weeks of age could represent maternal antibodies, as pigs from the second study without early-stage viremia did not show IgG antibodies at this age.

Several reports have demonstrated the immunomodulatory activity of PCV2, with a recent review providing an in-depth discussion of this topic [30]. However, limited information is available regarding PCV3. Zhang et al. (2020) demonstrated that PCV3 not only inhibits type I IFN signaling and the production of this pro-inflammatory cytokine via the cGAS-STING pathway [31] but also induces a cytokine storm, including TNF-α, IL-1b, IFN-γ, IL-6, and CCL5 [32]. These pro-inflammatory responses may contribute to the multiorgan inflammation observed in PCV3 infections [33]. In addition to infecting immune cells such as neutrophils and macrophages, PCV3-induced inflammation leads to lymphocyte dysplasia, necrosis, and disruption of the immune system [32]. These effects could increase susceptibility to secondary infections [32] and impair the immune response, potentially reducing antibody levels in oral fluids [34], particularly in cases of high PCV3 viral load and co-infection with PCV3.

This study’s main limitation is that it was conducted under commercial farm conditions, which, despite strict biosecurity measures, differ from controlled experimental settings. Another limitation is the use of Ct values for reporting viral DNA loads, as we were unable to quantify viral loads in terms of DNA copies per milliliter. Future research should focus on quantifying viral loads and investigating the immunological mechanisms of PCV2 and PCV3 co-infection to understand their clinical impact better and inform management practices.

## 5. Conclusions

This study assessed the effects of PCV2 and PCV3 co-infection on farm productivity and the antibody response to PCV2 vaccine. These results indicate that co-infection did not increase mortality, but it did provoke an abnormal IgG response in oral fluids. Further studies are needed to evaluate the impact of this antibody reduction in farms co-infected with other viruses, such as PRRSV or influenza, or bacteria like *Mycoplasma* spp. or *Actinobacillus pleuropneumoniae*.

## Figures and Tables

**Table 1:** PCV2 DNA in the serum of pigs aged 3 to 21 weeks.

Weeks of Age										
Pool	3	4	5	6	7	8	9	13	17	21
1	-	-	-	-	-	29.33	29.49	35.83	-	28.29
2	31.77 *	-	-	-	-	30.62	33.74	34.96	34.86	29.73
3	32.49	-	-	-	-	31.27	37.40	38.83	34.03	29.18
4	-	-	-	-	-	29.94	33.75	36.36	-	29.32
5	-	-	-	-	-	30.43	32.45	37.5	33.60	29.1
6	-	-	-	-	-	28.22	30.69	30.13	30.61	32.48
7	-	-	-	-	-	32.83	33.08	31.68	35.00	28.93

* Values represent the Ct. Ct values > 35 were considered negative.

**Table 2:** PCV2 DNA in the oral fluid of 3- to 21-week-old pigs.

	3	4	5	6	7	8	9	13	17	21
Pen 1	-	-	-	31.86 *	32.14	30.85	31.47	33.73	34.63	29.8
Pen 2	-	-	-	30.86	32.14	30.85	31.31	34.19	34.86	30.26
Pen 3	-	-	-	32.14	32.11	33.07	30.87	33.69	34.39	30.29
Pen 4	-	-	-	31.81	31.19	32.19	29.95	33.65	-	28.62
Pen 5	-	-	-	32.26	31.39	31.86	30.47	34.88	-	28.05
Pen 6	-	-	-	32.39	32.29	32.38	30.98	33.62	29.47	26.76

* Values represent the Ct. Ct values > 35 were considered negative.

**Table 3:** PCV3 DNA in sera from pigs aged 3 to 21 weeks.

Weeks of Age										
	3	4	5	6	7	8	9	13	17	21
Pool 1	25.66 *	-	32.33	30.95	30.86	25.42	30.58	32.73	32.64	26.12
Pool 2	-	-	-	-	-	25.59	28.69	30.85	31.62	27.96
Pool 3	-	-	-	-	-	26.50	33.70	33.89	33.67	26.74
Pool 4	-	-	-	-	32.55	29.50	27.31	32.95	33.05	26.74
Pool 5	34.60	-	-	-	35.45	26.27	27.74	31.1	33.45	26.72
Pool 6	-	-	-	-	-	27.83	30.17	27.66	30.95	27.69
Pool 7	-	31.00	-	-	35.30	27.35	31.64	32.66	34.09	26.13

* Values represent the Ct. Ct values > 35 were considered negative.

**Table 4:** PCV3 DNA in oral fluids from pigs aged 3 to 21 weeks.

	3	4	5	6	7	8	9	13	17	21
Pen 1	-	29.0 *	-	35.14	28.69	27.53	29.92	31.84	33.01	31.75
Pen 2	-	29.0	32.15	32.25	29.43	26.23	29.78	32.71	32.53	32.5
Pen 3	-	34.0	34.36	33.24	28.89	28.5	28.35	33.66	32.28	32.62
Pen 4	-	33.0	32.39	32.39	28.91	28.32	28.36	30.65	33.23	30.8
Pen 5	-	31.6	-	-	28.16	28.13	29.30	30.50	33.29	31.18
Pen 6	-	31.0	34.91	33.86	28.48	20.07	29.70	32.34	32.60	29.04

* Values represent the Ct. Ct values > 35 were considered negative.

**Table 5:** PCV2 and PCV3 DNA in oral fluids of pigs aged 3 to 11 weeks: a cross-sectional study.

	PCV2	PCV3
Week 3	-	-
Week 3	-	-
Week 5	-	-
Week 5	-	-
Week 7	-	-
Week 7	-	-
Week 9	-	-
Week 9	-	-
Week 11	32.48 *	34.09
Week 11	32.18	33.28

* Values represent the Ct. Ct values > 35 were considered negative.

**Table 6:** PCV2 DNA in the serum of pigs aged 3 to 21 weeks.

	3	4	5	6	7	8	9	13	17
Pig 36	-	-	-	-	-	30.41 *	30.74	35.29	-
Pig 37	-	-	-	-	-	28.69	32.47	36.64	32.42
Pig 38	-	-	-	-	-	29.53	31.69	-	-
Pig 39	-	-	-	-	-	29.85	33.75	37.27	-
Pig 40	-	-	-	-	-	30.65	34.42	38.92	-

* Values represent the Ct. Ct values > 35 were considered negative.

**Table 7:** PCV2 DNA in the serum of pigs aged 3 to 21 weeks.

	3	4	5	6	7	8	9	13	17
Pig 36	25.04 *	29.0	26.70	25.64	35.33	27.37	32.15	34.14	34.14
Pig 37	-	-	-	-	35.56	26.35	28.42	31.92	31.92
Pig 38	-	-	36.99	35.14	35.35	25.59	30.59	35.47	35.47
Pig 39	-	-	-	35.70	35.92	27.07	30.18	31.78	31.78
Pig 40	-	-	-	-	34.57	27.29	29.69	30.5	30.50

* Values represent the Ct. Ct values > 35 were considered negative.

## Data Availability

Data are contained within the article.
